# Low-Dose Melittin Enhanced Pigment Production Through the Upregulation of Tyrosinase Activity and Dendricity in Melanocytes by Limiting Oxidative Stress: A Therapeutic Implication for Vitiligo

**DOI:** 10.3390/antiox13111424

**Published:** 2024-11-20

**Authors:** Manoj Kumar Tembhre, Shipra

**Affiliations:** Department of Cardiac Biochemistry, All India Institute of Medical Sciences (AIIMS), New Delhi 110029, India; shipra9goel@gmail.com

**Keywords:** melittin, melanocyte, skin pigmentation, melanogenesis, tyrosinase, vitiligo, oxidative stress

## Abstract

Melittin is a major active ingredient of the bee venom produced by honeybees (*Apis mellifera*) that exerts various biological effects, such as anti-inflammatory, anti-tumor, anti-microbial, and antioxidant. The role of melittin in modulating melanin production by melanocytes is not known. Therefore, the present study aimed to study the effect of melittin on melanin production by human melanocytes along with its antioxidant status. Cultured human melanocytes were treated with melittin in a dose- and time-dependent manner, followed by the study of the cell viability, cell proliferation, and total melanin content. The effects of melittin in combination with narrow-band ultraviolet B (NB-UVB) on the total melanin content, melanocyte dendricity, oxidative stress, and the expression of genes associated with melanogenesis were investigated. An increased melanin content was observed with a low dose of melittin (LDM) (alone or in combination with NB-UVB), and there was a corresponding increase in the tyrosinase activity, melanocyte dendricity, and melanogenesis-associated genes. The present study concluded that LDM alone or LDM (+NB-UVB) can induce melanin synthesis by increasing the tyrosinase activity in melanocytes by limiting the oxidative stress, and this may be therapeutically exploited as an adjuvant therapy for vitiligo.

## 1. Introduction

Human skin is the largest organ of the body, and skin pigment, i.e., melanin, plays an important role in providing protection against harmful ultraviolet radiation (UVR) [1]. The type, distribution, and amount of melanin play important roles in determining the colors of the skin, hair, and eyes, and these attributes generally vary with the ethnicity and genetic constitution of an individual. Melanin is a complex mixture of pigmented indole-rich biopolymers that are synthesized by specialized cells called melanocytes, located in the basal layer of the epidermis of the skin. There are two main types of melanin polymers, i.e., eumelanin (black/brown, highly polymerized) and pheomelanin (yellow/reddish-brown, less polymerized, and sulfur-rich) [2]. The synthesis, storage, and transport of pigment occur through specialized organelles called melanosomes, present in the melanocytes, and this process is regulated by an intricate network of proteins (e.g., tyrosinase (TYR)—a rate-limiting enzyme in melanogenesis; tyrosinase-related protein 1/2 (TYRP1/2); oculocutaneous albinism type 2 (OCA2); solute carrier family 45 member 2 (SLC45A2); premelanosome protein (PMEL17) or gp100; paired box gene 3 (PAX3), alpha-melanocyte-stimulating hormone (α-MSH); ras-related protein-27A (RAB27A), etc.).

The process of melanin synthesis is a potential source of reactive oxygen species (ROS), and melanocytes are under the influence of continuous endogenous (e.g., mitochondria, peroxisomes, cytochrome P450, NAPDH oxidases, etc.) and exogenous (e.g., ionizing radiation, UVR, environmental toxins, etc.) oxidative insults. However, melanocytes have a robust antioxidant defense system that can rigorously overcome ROS insults. The dysregulation of the fine-tuning of the ROS (oxidant)–antioxidant defense mechanism may lead to excessive oxidative stress, thereby endangering the survival of melanocytes and eventually causing melanocyte cytotoxicity [3,4], as reported in vitiligo.

Vitiligo is considered an autoimmune depigmenting disease that is clinically characterized by the patchy loss of skin pigment (melanin) due to the selective killing of melanocytes. A multifactorial etiology has been postulated to explain the pathogenesis of vitiligo that involves neural, biochemical, genetic, and immunological components culminating in melanocyte cytotoxicity. Cytotoxic CD8+T cells were considered as culprit cells along with a deficiency of regulatory T cells [3,5,6] in the pathogenesis of vitiligo. At present, there is no cure for vitiligo, and available treatment modalities include pharmacology (e.g., topical and oral corticosteroid, calcineurin inhibitors, azathioprine, methotrexate, other systemic therapies), photochemotherapy/phototherapy (e.g., narrow-band ultraviolet B (NB-UVB), PUVA (psoralen and UVA)/PUVAsol), and surgical interventions (e.g., autologous transplantation of blister skin graft and non-cultured epidermal cell suspension for stable vitiligo). NB-UVB is one of the most promising phototherapies in vitiligo, but its clinical efficacy is also limited, as not all patients equally respond to the treatment [7,8,9].

Therefore, alternative treatment strategies that may lead to repigmentation in the vitiligo lesional skin are highly warranted. With this aim, we identified melittin, which is a major active ingredient of the bee venom produced by all species of honeybees. Melittin is a twenty-six-amino-acid-long peptide, and it possesses anti-inflammatory, anti-tumor, anti-microbial, and anti-parasitic properties along with antioxidant properties [10,11,12,13]. The role of melittin in regulating melanin synthesis by melanocytes has not been investigated yet. In the present study, we investigated the effect of melittin on melanin production in the presence or absence of NB-UVB in cultured human melanocytes, followed by determining the expression of the key factors involved in melanogenesis and melanin transfer along with the status of oxidative stress in melanocytes.

## 2. Materials and Methods

### 2.1. Isolation and Culture of Melanocytes from Human Skin

In the present study, we collected foreskin (i.e., surgically discarded Fitzpatrick skin type IV) samples from the Department of Surgery, AIIMS, New Delhi, after receiving informed consent from the donors (who were not suffering from any autoimmune, inflammatory, or infectious diseases and had not taken any medication in the past three months prior to sample collection) who underwent circumcision. Samples were collected and transported in a transportation medium (50 mL HBSS containing 100 μL gentamycin sulphate, 50 μL amphotericin (Gibco Ltd., Grand Island, NY, USA), 500 μL penicillin–streptomycin (Gibco Ltd., Grand Island, NY, USA)) on ice. The skin tissue was rinsed with fresh HBSS medium (Gibco Ltd., Grand Island, NY, USA) once, followed by a quick wash with 70% ethanol and a second wash with HBSS. The skin was then incubated with Dispase II (Roche Ltd., Basel, Switzerland) for 14–15 h at 4 °C to separate the dermis from the epidermis. The epidermal layer was removed with the help of sterile forceps and kept inverted (the dermis side facing up, to prevent drying) in HBSS (Gibco Ltd., Grand Island, NY, USA). The epidermal cell suspension was incubated in 0.1% Trypsin/EDTA (Gibco Ltd., Grand Island, NY, USA) solution at 37 °C for 10–15 min and vortexed intermittently. Trypsin was neutralized with HBSS followed by 1000× *g* for 10 min. The supernatant was discarded, and cells were plated in M254 medium supplemented with growth factors (Human Melanocyte Growth Supplement (HMGS), Gibco Ltd., Grand Island, NY, USA) and 1X antibiotic–antimycotic (penicillin, streptomycin, and amphotericin-B) solution (Himedia Laboratories, Maharashtra, India), then incubated at 37 °C 5% CO_2_ for two days. Cells were fed with M254 medium daily until they were populated with melanocytes. Repeated trypsinization killed the contaminating keratinocytes, and fibroblasts were removed by treating the cells with 100 µg/mL geneticin (Sigma-Aldrich Ltd., St. Louis, MO, USA) for three consecutive days. Cells were passaged when the cell density was >80% using trypsin. Cells were cryopreserved in liquid nitrogen in freezing medium (Promocell Ltd., Heidelberg, Germany) and thawed for subsequent experiments according to the manufacturer’s protocol.

### 2.2. Melittin and NB-UVB Treatment of Cultured Melanocytes

Melanocytes were plated at a seeding density of 2–5 × 10^5^ cells in 6- or 24-well plates and treated with different concentrations (i.e., 0.1 µM, 1 µM, 2 µM, 5 µM, and 10 µM) of melittin at 80% confluency for different time intervals (i.e., 0 h, 24, 48 h, 72 h, 96 h). Melittin (twenty-six residue polypeptide, Cat. #444605 (CAS # 37231-28-0), empirical formula: C_131_H_229_N_39_O_31_, assay purity ≥97%, Calbiochem Ltd., San Diego, CA, USA) is hydrophilic in nature and readily dissolved in water. We dissolved the melittin in sterile water, and the control received an equal volume of sterile water (vehicle) that served as the negative control in all experiments. In experiments where melittin (0.1 µM for 48 h) was given in combination with NB-UVB, the melanocyte-cultured plates (wrapped in transparent polythene to avoid contamination) were irradiated once with 0.28 Jcm^−2^ of NB-UVB radiation using an NB-UVB chamber (UV7001 Waldmann, H. Waldmann GmbH & Co. KG, Villingen-Schwenningen, Germany) for 5 min (throughout the experiments, the melanocytes were kept in M254 medium with growth factors and other supplements except for NB-UVB irradiation, where the cells were maintained in Dulbecco’s phosphate buffered saline (Sigma-Aldrich Ltd., St. Louis, MO, USA) without any growth factors and supplements). Post NB-UVB irradiation, the media was replaced with complete media (as mentioned above), and the cells were allowed to grow for 6 h followed by melittin treatment. Depending on the experiments, the cells were harvested and used for further studies.

### 2.3. Cell Viability and Proliferation Assay

The cell viability and proliferation assay was performed using an MTT (3, 4, 5 dimethylthiazol 2, 2, 5, diphenyl tetrazolium bromide) assay kit. Primary human melanocyte-cultured cells were cultured in a 96-well culture plate (50,000 cells/well) with M254 culture media. An MTT assay was performed using an MTT assay kit (Sigma-Aldrich, St. Louis, MO, USA) at 0 h, 24 h, 48 h, 72 h, and 96 h, at different melittin concentrations (0.1 µM, 1 µM, 2 µM, 5 µM, and 10 µM). Then, 10 µL of an MTT reagent (0.5 mg/mL in PBS) was added to the wells in triplicate and incubated for 3.5 h at 37 °C in a humidified atmosphere. After incubation, the medium was removed, 100 µL of solubilization solution (dissolves formazan crystals) was added to the wells, and the absorbance was measured at 570 nm by an ELISA microplate reader (iMark Microplate Reader, Bio-Rad Ltd., Hercules, CA, USA). All the steps were performed as per the manufacturer’s instructions (average values from triplicate readings were determined, with the average value subtracted from the blank. The absorbance was plotted against the number of cells/mL. The number of cells used in the assay lay within the linear portion of the plot and yielded an absorbance of 0.75–1.25). The cell viability was expressed in percentages, and the cell proliferation was presented relative to the control.

### 2.4. Melanin Content, Tyrosinase Activity Assay, and Melanocyte Dendricity Analysis

The melanin content of the melanocytes was determined in the cell lysates using a human (Sandwich) Melanin ELISA kit (ABIN577604, antibodies-online GmbH Ltd., Berlin, Germany), according to the manufacturer’s instructions provided in the kit (ABIN577604, detection range 12.5–1000 pg/mL). Detection was performed at a wavelength of 450 nm by the colorimetric method using the ELISA microplate reader (iMark Microplate Reader, Bio-Rad Ltd., Hercules, CA, USA). As per the manufacturer’s instruction, a standard curve was constructed by plotting the mean absorbance for each standard on the *x*-axis against the concentration on the *y*-axis, and a best fit curve was drawn through the points on the graph. The unknown concentration was determined by extrapolating the curve. The tyrosinase activity was determined using a one-step Tyrosinase Activity Assay kit (sensitivity = 30 μU, Abcam Ltd., Cambridge, UK). Detection was performed at wavelength of 510 nm by the colorimetric method. All the steps were performed as per the manufacturer’s instructions. The melanocyte dendricity was determined by manually counting the dendrites per melanocytes in triplicate (melanocytes with greater than two dendrites were only counted in the measurement in three different fields, 20×) using an Eclipse Ts2 inverted microscope (Nikon Ltd., Tokyo, Japan).

### 2.5. Gene Expression Analysis by qPCR (Quantitative Polymerase Chain Reaction)

As previously described [5], the transcript expression analysis was performed by qPCR for melanin synthesis- and melanin transfer-associated genes using specific primers purchased from Sigma-Aldrich (genes and their forward and reverse primer sequences are listed in Table 1). Briefly, according to the manufacturer’s instructions, TRIzol^TM^ (ThermoFisher Scientific, Carlsbad, CA, USA) reagent was used to isolate the total RNA from the cultured melanocytes. An RNase free–DNase I kit (ThermoFisher Scientific, Carlsbad, CA, USA) was used to remove the contaminating DNA followed by complementary DNA (cDNA) synthesis using the RevertAid first strand cDNA synthesis kit (ThermoFisher Scientific, Carlsbad, CA, USA). The qPCR reaction was set as per the manufacturer’s instructions using the Maxima SYBR Green qPCR Master Mix (ThermoFisher Scientific) in a CFX96 Real-time PCR System (BioRad, Hercules, CA, USA). The PCR settings used were an initial denaturation at 95 °C for 10 min, followed by 40 cycles of 15 s of denaturation at 95 °C and 30 s of primer annealing at 60 °C, with an extension at 72 °C. The reactions were run in duplicate (average Ct values were used for analysis), and β-actin served as the internal control for normalization. The relative gene expression data were expressed as fold change (2^−ΔΔCt^).

### 2.6. Analysis of Oxidative Stress in Cultured Melanocytes

The harvested cells were processed as per the manufacturer’s instructions, and the status of oxidative stress was determined using an 8-hydroxy 2 deoxyguanosine (8-OHdG) assay kit (ab201734, Abcam Ltd., Cambridge, UK), a malondialdehyde (MDA) assay kit (ab118970, Abcam Ltd., Cambridge, UK), a total glutathione (GSH+GSSG)/reduced glutathione (GSH) assay kit (ab239709, Abcam Ltd., Cambridge, UK), a Superoxide Dismutase (SOD) activity assay kit (E-BC-K019-M, Elabscience Biotechnology Inc., Houston, TX, USA), and a Catalase activity assay kit (E-BC-K031-M, Elabscience Biotechnology Inc., Houston, TX, USA) in the treated (as explained above) cultured melanocytes. The colorimetric detection method was used in all assays.

### 2.7. Statistical Analysis

All statistical analyses were performed, and graphs were generated using STATA software version 14 (StataCorp LP, College Station, TX, USA) and Graph Pad Prism version 5 (San Diego, CA, USA). In the present study, the multiple groups were analyzed by one/two-way ANOVA (analysis of variance). A *p* value of < 0.05 was set as significant.

## 3. Results

**Figure 1:**
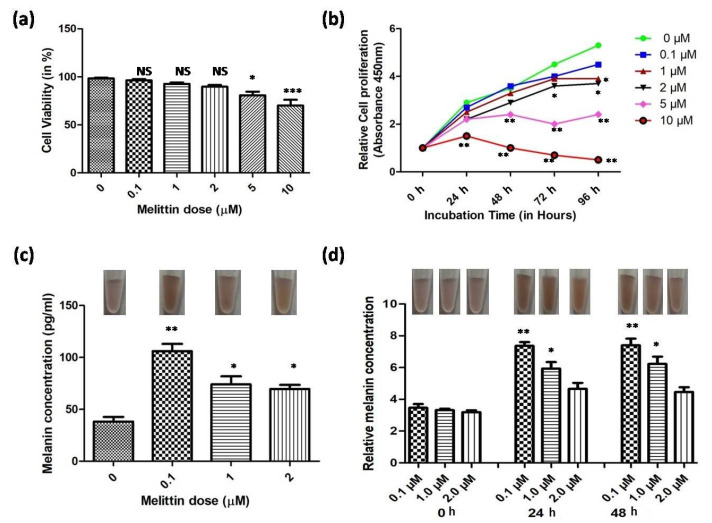
Effect of melittin (dose- and time-dependent) on cell viability, cell proliferation, and melanin content of cultured human melanocytes. (**a**) Cell viability in percentage at different concentrations of melittin (0.1 µM, 1 µM, 2 µM, 5 µM, and 10 µM) after 24 h; (**b**) relative cell proliferation at different concentrations of melittin (0.1 µM, 1 µM, 2 µM, 5 µM, and 10 µM) and at different time points (0 h, 24 h, 48 h, 72 h, 96 h); (**c**) melanin content (pg/mL) in melittin- (0.1 µM, 1 µM, 2 µM) treated melanocytes (24 h) (upper panel shows the representative melanocyte cell pellets in suspension); (**d**) relative melanin content of cultured melanocytes treated with different concentrations of melittin at different time points (* *p* < 0.01; ** *p* < 0.001; *** *p* < 0.0001; NS = not significant).

We tested the effect of melittin on the cell viability at different doses, i.e., 0.1 µM, 1 µM, 2 µM, 5 µM, and 10 µM, where we found no significant difference in the cell viability up to a concentration of 2 µM compared to the control but a significantly decreased cell viability reported at 5 µM and 10 µM (Figure 1a). Further, when we studied the dose- (0.1 µM, 1 µM, 2 µM, 5 µM, and 10 µM) and time- (0 h, 24 h, 48 h, 72 h, and 96 h) dependent effect on cell proliferation, we observed no significant difference on the cell proliferation for melittin concentrations of 0.1 µM, 1 µM, and 2 µM, at 24 h and 48 h time intervals (Figure 1b). A sharp decline in the cell proliferation ability was reported after 48 h, i.e., 72 h and 96 h (Figure 1b). Based on the cell viability and proliferation assay, the cut-off for LDM (low melittin dose) was considered as 2 µM, where no significant difference was observed in the cell viability. Therefore, we limited our investigation to up to a 2 µM melittin concentration in the successive experiments. Next, we determined the total melanin content (pg/mL) of the melanocytes treated with melittin (0.1 µM, 1 µM, 2 µM) for 48 h, where we found the peak melanin concentration (i.e., 106.01 ± 12.7 vs. control (38.33 ± 7.64)) at low-dose (i.e., 0.1 µM, *p* < 0.001), and melanin concentration remained significantly higher (*p* < 0.01) at the melittin dose i.e., 1 µM (74.1 ± 13.45) and 2 µM (69.67 ± 6.81) compared to the controls (38.33 ± 7.64) (Figure 1c). Similar trends were found when we compared the relative melanin content, and the maximum response was observed at LDM, 0.1 µM for 24 h and 48 h (*p* < 0.001) (Figure 1d). Considering the above observations, we limited our study to LDM (i.e., 0.1 µM) for 48 h for subsequent experiments, since peak response in the melanin content was observed at 0.1 µM LDM, and we used the term LDM for this dose.

**Figure 2:**
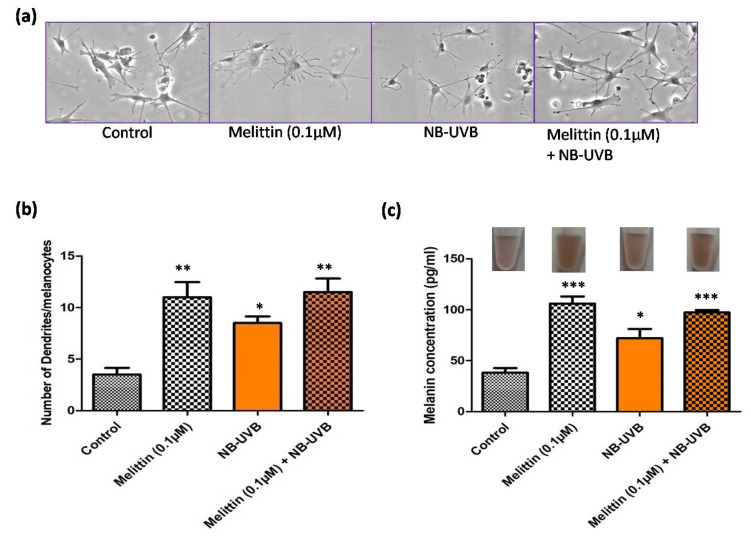
Effect of melittin and narrow-band ultraviolet B (NB-UVB) on the melanocytes’ dendricity and melanin content of cultured human melanocytes. (**a**) Representative light field images (20×) of cultured melanocytes in the absence or presence of melittin (0.1 µM), NB-UVB (0.28 Jcm^−2^) and the combination of melittin and NB-UVB after 48 h of culture; (**b**) number of dendrites per 100 melanocytes in the absence or presence of melittin (0.1 µM), NB-UVB (0.28 Jcm^−2^) and the combination (melittin + NB-UVB) after 48 h of culture; (**c**) melanin content (pg/mL) in cultured melanocytes (upper panel shows the representative melanocyte cell pellets in suspension) with or without melittin (0.1 µM) or NB-UVB (0.28 Jcm^−2^) or the combination of both (melittin + NB-UVB) after 48 h of culture. (* *p* < 0.01; ** *p* < 0.001; *** *p* < 0.0001).

As mentioned above, a previous study reported increased melanogenesis with NB-UVB treatment, and NB-UVB based phototherapy has been widely used in the treatment of vitiligo; therefore, we extended our study to compare the effect of LDM and NB-UVB (0.28 Jcm^−2^) alone (LDM) or in combination (LDM+NB-UVB). We observed a significantly increased dendricity in the melanocytes treated with LDM (*p* < 0.001) and NB-UVB (*p* < 0.01) compared to the controls (Figure 2a,b). The effect on melanocyte dendricity was found to be comparable between LDM alone and the combination of LDM+NB-UVB. A similar trend was observed for the total melanin concentration, where the magnitude was found to be comparable between LDM and the combination dose compared to the control (*p* < 0.0001) (Figure 2c). The melanin content was also found to be significantly higher in melanocytes treated with NB-UVB alone (*p* < 0.01), but the magnitude was lower than LDM alone and the combination dose.

**Figure 3:**
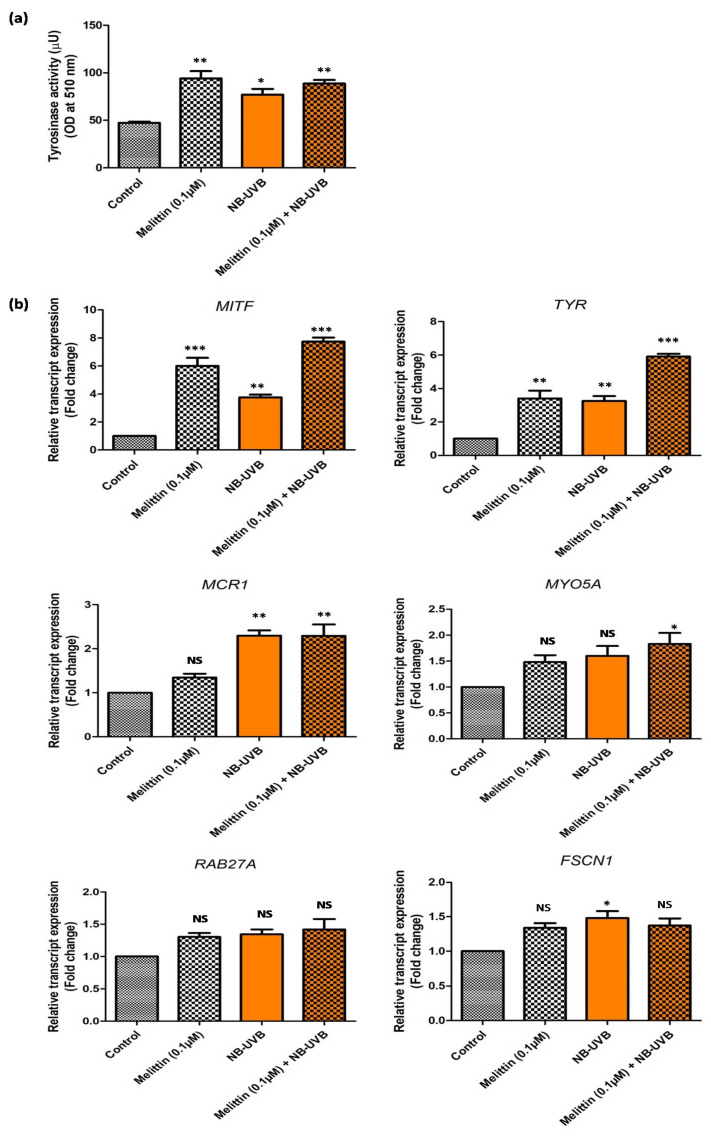
Effect of melittin and NB-UVB on (**a**) tyrosinase activity of melanocytes; (**b**) transcript (mRNA) expression of genes associated with melanin synthesis and melanin transfer in cultured human melanocytes treated with melittin (0.1 µM), NB-UVB (0.28 Jcm^−2^), and combination (melittin + NB-UVB) after 48 h of culture. (* *p* < 0.01; ** *p* < 0.001; *** *p* < 0.0001; NS = not significant).

**Figure 4:**
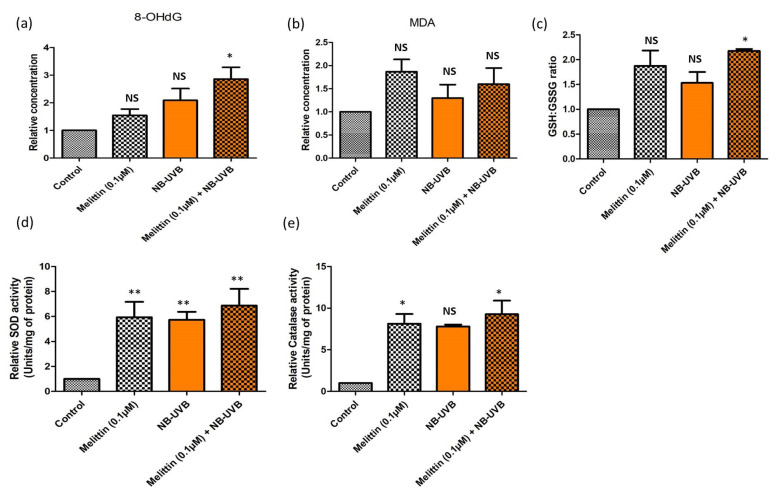
Effect of melittin and NB-UVB on oxidative stress-associated markers: (**a**) 8-hydroxy-2’-deoxyguanosine (8-OHdG); (**b**) malondialdehyde (MDA); (**c**) ratio of total glutathione (GSH+GSSG)/reduced glutathione (GSH); (**d**) catalase activity; (**e**) superoxide dismutase (SOD) activity, of cultured human melanocytes treated with melittin (0.1 µM), NB-UVB (0.28 Jcm^−2^), and combination (melittin + NB-UVB) after 48 h of culture. (* *p* < 0.01; ** *p* < 0.001; NS = not significant).

In a similar experimental set-up, we determined the tyrosinase activity of melanocytes; the effect of LDM alone and the combination dose was found to be comparable, but it was significantly higher (*p* < 0.001) in both groups compared to the control (Figure 3a). The tyrosinase activity was also significantly increased (*p* < 0.01) in the melanocytes treated with NB-UVB compared to the control (Figure 3a). Next, we studied the expression (transcript) level of genes associated with melanogenesis and melanin transfer in all three groups (Figure 3b). We observed a significantly increased expression of *MITF* (microphthalmia associated transcription factor) and *TYR* (tyrosinase) in all three groups compared to the controls with the maximum expression in the combination dose. However, no significant difference (*p* > 0.05) was observed in the expression level of *MCR1* (melanocortin receptor 1) on melanocytes treated with LDM alone compared to the controls, but increased expression was observed in the NB-UVB and combination groups (*p* < 0.001) compared to the control (Figure 3b). We also studied the expression of three genes that were associated with melanin transfer from melanocytes by exocytosis, where no significant difference (*p* > 0.05) was observed for *MYO5A* (Myosin-Va 5A) (except for the combination group, where the *MYO5A* expression was significantly higher compared to the control (*p* < 0.01), *RAB27A* (Ras-related protein 27A), and *FSCN1* (fascin actin-bundling protein) (except for the NB-UVB group, where the *FSCN1* expression was significantly upregulated compared to the control (*p* < 0.01) (Figure 3b). We further investigated the effect of LDM and NB-UVB alone or in combination on the status of oxidative stress in melanocytes using hallmark markers. No significant difference (*p* > 0.05) was observed in the levels of 8-hydroxy-2’-deoxyguanosine (8-OHdG) (except for the combination group, where the concentration of 8-OHdG was increased compared to the control (*p* < 0.01)) (Figure 4a), malondialdehyde (MDA) (Figure 4b), and the ratio of total glutathione (GSH+GSSG)/reduced glutathione (GSH) (Figure 4c) (except for the combination group, where the ratio of GSH+GSSG/GSH was higher compared to the control (*p* < 0.01)). However, the catalase activity remained significantly high in all the three groups compared to the control (*p* < 0.001) (Figure 4d), and no significant difference was observed in the activity of superoxide dismutase (SOD) in the NB-UVB group compared to the control. The SOD activity (Figure 4e) was found to be increased in the LDM and combination groups (*p* < 0.01) compared to the control.

## 4. Discussion

**Figure 5:**
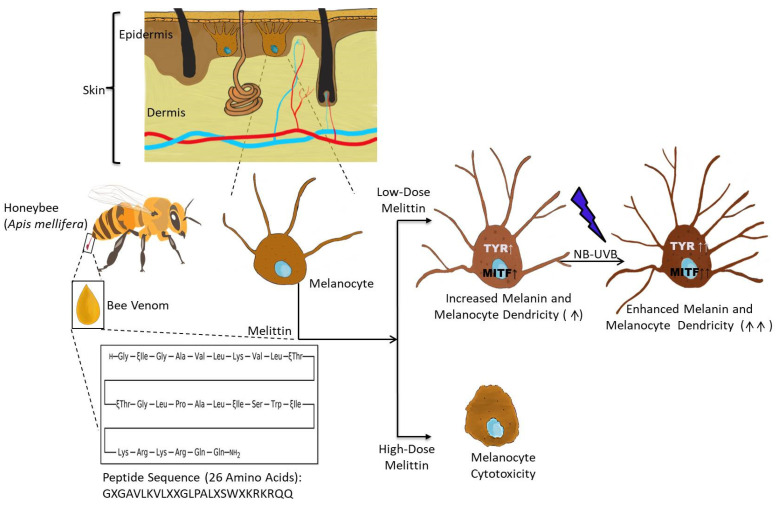
Schematic representation of the mechanism of action of low-dose melittin (LDM) (active ingredient of bee venom produced by honeybees) and NB-UVB in enhancing the melanin synthesis (by upregulating the activity of rate-limiting enzyme tyrosinase (TYR), transcription factor *MITF,* and total melanin content) and melanocyte dendricity. The high dose of melittin leading to melanocyte cytoxicity may be contributed by the lytic properties of melittin and/or enhanced oxidative stress (note: the upward arrow indicates the increased expression, and the number of arrows indicates the magnitude of the increase).

As mentioned above, melanin is an essential component of skin, imparting different colorations to the skin based on the type and constitution of the melanin. It is synthesized by melanocytes, packaged into specialized vesicles called melanosomes, and transferred to the neighboring keratinocytes present in the epidermis of the skin. The process of melanin synthesis (melanogenesis) and melanin transfer occurs in a highly regulated manner involving myriad proteins/transcription factors. As described earlier, in vitiligo, there is a spontaneous loss of melanin from the skin due to the selective killing of melanocytes, and current treatment modalities are largely confined to immunosuppressives, phototherapies, and surgical interventions with limited efficacies. Aside from vitiligo, there are various other acquired pigmentation-associated diseases like melanoma and melasma. Melittin, a major component of bee venom, accounts for ~40–50% of the dry weight of venom [14]. Structurally, it is a small twenty-six amino acid linear cationic peptide, and it interacts with negatively charged cell membranes by electrostatic bonding causing the destabilization/disruption of the membrane via pore formation [15,16]. Owing to its lytic cytotoxic effect, the role of melittin has been widely explored in different types of cancer cells [17]. However, melittin also possess potent antioxidant properties and confers cell protective effects [13,18]. The antioxidant property prompted us to explore the effect of melittin on melanocyte function, particularly melanin production. We investigated the dose- and time-dependent effect of melittin on melanocyte cell survival and proliferation and found that the melanocyte viability was not affected at 0.1 µM, 1 µM, and 2µM concentrations of melittin, and no significant difference was observed in the cell proliferation capacity of melanocytes at 24 h and 48 h for these concentrations of melittin. A significant decrease in the cell viability and proliferation potential of melanocytes was reported at 5 µM and 10µM. These findings suggested that LDM did not affect the cell viability and proliferation, but at high doses, it was found to be detrimental for cells. Further, we observed a peak melanin concentration at LDM (0.1 µM) when cultured for 48 h. Therefore, we limited our focus to LDM for 48 h in the subsequent experiments. Increased melanocyte dendricity and melanin content were observed in LDM, NB-UVB, and the combination group compared to the control, and the magnitude of these parameters remained higher in LDM and the combination group compared to the NB-UVB group indicating a better efficacy of LDM alone or when combined with NB-UVB. We observed a similar trend for tyrosinase activity, suggesting the direct effect of melittin on tyrosinase, which is a rate-limiting enzyme in melanogenesis. The gene expression data also corroborated the above findings, where the mRNA expression levels of *TYR* and *MITF* were found to be increased in LDM, but no effect of LDM was observed on *MCR1*, *MYO5A*, *RAB27A*, and *FSCN1.* These observations suggested that LDM increased the melanin synthesis (melanin content) by upregulating the tyrosinase activity and transcription factor *MITF*. The expression of genes associated with melanin transfer was not affected by LDM; this may be due to the culture of isolated melanocytes, and these genes may require priming by keratinocytes. The summary of the outcome of the present study has been graphically explained in Figure 5. In human skin, each melanocyte is surrounded by ~30–40 keratinocytes, and this association is termed an ‘epidermal melanin unit’ [19]. In melanocytes, melanins are packed in membrane-bound ‘melanosomes’, the transfer of this cargo takes place along the dendrites of melanocytes to keratinocytes by cell-to-cell contact/exocytosis, and the keratinocyte uptake of melanosome occurs through endocytosis. Therefore, to gain further insight, investigation with co-culture of melanocyte–keratinocyte is required to understand the precise mechanism of action of LDM on melanin transfer, which will be the subject of our future investigations. Since melanogenesis generates ROS and requires oxidant–antioxidant balance, the effect of melittin (antioxidant effect) on the oxidative stress of melanocytes is highly desired. Considering these notions, we investigated the hallmark biomarkers of oxidative stress, where no significant increase in 8-OHdG, MDA, and the GSH+GSSG/GSH ratio was observed in LDM group, but the levels of ROS scavenging enzymes, i.e., catalase and SOD, were significantly elevated in the LDM group compared to the control. These findings supported the antioxidant properties of melittin (LDM) and its function as a potent inducer of melanogenesis. However, further investigation is highly warranted using robust experimental models to test the in vivo efficacy of LDM in inducing melanogenesis and to support the therapeutic implication of LDM in the management of vitiligo.

## 5. Conclusions

The present study demonstrated the potential of melittin (i.e., LDM) in regulating the melanogenesis process in human melanocytes. LDM can also be considered as adjuvant therapy in vitiligo and can be given in conjunction with NB-UVB to restore the pigmentation in the vitiliginous skin of vitiligo patients. The in vivo validation of our preliminary findings is highly warranted, thereby limiting its instant translational implications.

## Figures and Tables

**Table 1:** List of genes and their respective forward and reverse primers.

Gene	Gene Accession Number	Primer Sequence Forward Primers (5′-3′) Reverse Primers (5′-3′)
*βeta-ACTIN*	NM_003088.4	GCGTGACATTAAGGAGAAG GAAGGAAGGCTGGAAGAG
*MITF*	NM_006722.3	CAGACCTATTCCGCTCCATCTC CTTATCGGAGGCTTGGAGGC
*TYR*	NM_000372.5	GAAGGCACCGTCCTCTTCAA AGAGTCTGGGTCTGAATCTTGT
*MC1R*	NM_002386.4	CGAAATGTCCTGGGGACCTG GGGCTCAGGGATTCTCACAA
*MYO5A*	NM_000259.3	GTGAGCGAGGAGCTTGATGT TGGGTTGGATGGCCTCTTTC
*RAB27A*	NM_183236.3	GCAAGGTTGTGGAGAAAAGCA CCCTACACCAGAGTCTCCCA
*FSCN1*	NM_003088.4	CTGTCTGCCAATCAGGACGA CACTTTTTGGTGTCGCGGTC

*MITF*—microphthalmia associated transcription factor; *TYR*—tyrosinase; *MCR1*—melanocortin receptor 1; *FSCN1*—fascin actin-bundling protein; *MYO5A*—myosin-Va 5A; *RAB27A*—ras-related protein Rab-27A.

## Data Availability

We provided all the relevant information in the manuscript. Additional information may be obtained from the corresponding author through given email.
